# The landscape of bispecific T cell engager in cancer treatment

**DOI:** 10.1186/s40364-021-00294-9

**Published:** 2021-05-26

**Authors:** Shujie Zhou, Mingguo Liu, Fei Ren, Xiangjiao Meng, Jinming Yu

**Affiliations:** 1 Cheeloo College of Medicine, Shandong University, Jinan, Shandong China; 2 Department of Radiation Oncology, Shandong Cancer Hospital affiliated to Shandong First Medical University, Jinan, Shandong China; 3 Department of Oncology, Yuncheng Honesty Hospital, Heze, Shandong China; 4 Department of Radiation Oncology, Shandong Cancer Hospital affiliated to Shandong University, Jinan, Shandong China

**Keywords:** Immunotherapy, Bispecific T cell engager, Cancer

## Abstract

T cell-based immunotherapies have revolutionized treatment paradigms in various cancers, however, limited response rates secondary to lack of significant T-cell infiltration in the tumor site remain a major problem. To address this limitation, strategies for redirecting T cells to treat cancer are being intensively investigated, while the bispecific T cell engager (BiTE) therapy constitutes one of the most promising therapeutic approaches. BiTE is a bispecific antibody construct with a unique function, simultaneously binding an antigen on tumor cells and a surface molecule on T cells to induce tumor lysis. BiTE therapy represented by blinatumomab has achieved impressive efficacy in the treatment of B cell malignancies. However, major mechanisms of resistance to BiTE therapy are associated with antigen loss and immunosuppressive factors such as the upregulation of immune checkpoints. Thus, modification of antibody constructs and searching for combination strategies designed to further enhance treatment efficacy as well as reduce toxicity has become an urgent issue, especially for solid tumors in which response to BiTE therapy is always poor. In particular, immunotherapies focusing on innate immunity have attracted increasing interest and have shown promising anti-tumor activity by engaging innate cells or innate-like cells, which can be used alone or complement current therapies. In this review, we depict the landscape of BiTE therapy, including clinical advances with potential response predictors, challenges of treatment toxicity and resistance, and developments of novel immune cell-based engager therapy.

## Introduction

T cell-based cancer immunotherapies have transformed the clinical practice of cancer treatment by targeting and mobilizing T cells to eradicate malignant cells. Depending on the mechanisms of action, T cell-based cancer immunotherapies can be mainly divided into two classes: one against immunosuppressive factors represented by immune checkpoint inhibitors (ICIs), the other one focusing on immunostimulatory pathways represented by chimeric antigen receptor (CAR) T cells and T-cell engaging bispecific antibodies (bsAbs) [1–3]. ICIs have revolutionized cancer treatment in the clinic, especially in several advanced solid tumors, for instance, melanoma and non-small-cell lung cancer [4–7]. They hamper the tumor immune escape by blocking key immunosuppressive molecules such as programmed cell death 1 (PD-1) and its ligand (PD-L1) and releasing the “brake” of cytotoxic T cells to eliminate tumor cells, however, response rates of ICIs remain limited [8]. A major reason for this is the lack of a sufficient number of tumor-infiltrating immune cells (TILs), primarily T cells, in the tumor site, which is referred to as exhibiting cold phenotype [9]. CAR T-cell therapy is a newly developed adoptive cell therapy by genetically engineering T cells to express a CAR comprising intracellular T-cell signaling domains and an extracellular antigen-recognition structure targeting tumor-associated antigens (TAAs), redirecting and activating T cells to eradicate malignant cells specifically [10]. The preparation of CAR T Cells primarily includes isolation of T cells from patients, genetic modification of T cells, expansion of T cells in vitro, and infusion of edited T cells to patients, however, which is a complex and time-consuming process [11]. The other alternative approach to redirect T cells against target cells is T-cell engaging bsAbs with unique function engaging TAAs on cancer cells and cell surface molecules on T cells. Bispecific T-cell engager (BiTE) stands out as a novel subclass of T-cell engaging bsAbs with promising clinical results in the treatment of cancers. And the comparison of these three T-cell based immunotherapies is summarized in Table 1.

**Table 1:** Comparison of three main T cell-based immunotherapies: ICI, CAR T cell, and BiTE

	ICI	CAR-T cell	BiTE
Structure	Monoclonal antibody targeting immune checkpoint proteins	A T cell from patients that are genetically re-engineered to present a synthetic transmembrane receptor on the surface to target a tumor antigen on tumor cells	A recombinant antibody comprised of two tandem scFv, one binding CD3 on T cells, the other targeting a tumor antigen on tumor cells
Anti-tumor mechanisms	Blocking the inhibitory immune checkpoint proteins that result in cytotoxic T cell-mediated immune response	Inducing tumor cell lysis by the formation of immune synapse between T cells and tumor cells	Inducing tumor cell lysis by the formation of immune synapse between T cells and tumor cells
Recruitment of T cells	Passive, acting on tumor-infiltrating and endogenous T cells to kill tumor cells	Active, redirecting engineered T cells outside of body to kill tumor cells	Passive, dependent on endogenous T cells and redirecting them to kill tumor cells
Production	Hybridoma technology, pervasive for all patients	Genetically engineering patient’s T cells outside of body, individualized for each patient	Genetically engineered and purified from mammalian cell lines, pervasive for all patients
Indications	Mainly in solid tumors with approval in a small part of hematologic neoplasms	All in hematologic neoplasms	All in hematologic neoplasms but with promising results for solid tumors
Toxicity	Immune-mediated AEs such as hyperactivation and hypersensitivity	On-target off-tumor effects, CRS, neurotoxicity	On-target off-tumor effects, CRS, neurotoxicity
Advantages	Broad-spectrum anti-tumor activity, easy production	MHC-independent, TCR-independent, endogenous T cell-independent	MHC-independent, TCR-independent, relatively easy production, tumor-infiltrating T cell-independent
Disadvantages	Tumor-infiltrating T cell-dependent, immune checkpoint expression-dependent, MHC-dependent, TCR-dependent	Lack of efficacy for solid tumors, long-term and complex production, antigen-dependent	Antigen-dependent, continuous administration due to a short half-life

*ICI* Immune checkpoint inhibitor, *CAR* Chimeric antigen receptor, *BiTE* Bispecific T cell engager, *AEs* Advert effects, *CRS* Cytokine release syndrome, *MHC* Major histocompatibility complex, *TCR* T cell receptor

## BiTE design and mechanism of action

**Fig. 1:**
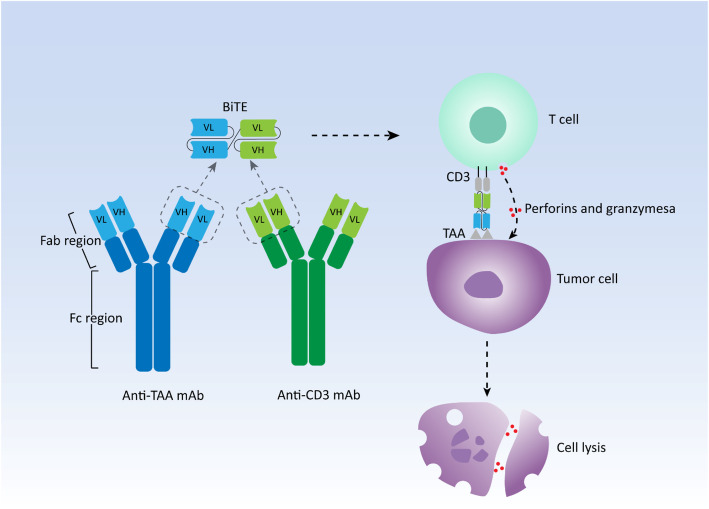
The schematic representation of structure and mechanism of action of canonical bispecific T-cell engager (BiTE). mAb: Monoclonal antibody; VH: Heavy chain variable region; VL: Light chain variable region; TAA: Tumor-associated antigen

In general, human antibodies are monospecific that usually recognize only one targeted antigen. bsAbs simultaneously target two different antigens for cancer treatment via redirecting immune cells to tumor cells, delivering drugs to tumors, and blocking two biological pathways significant for tumors [12]. Among them, redirecting immune cells, primarily T cells, to target cells is the most successful and widely used function to induce specific and powerful anti-tumor activity. Due to the invariant property of CD3 chains in the T cell receptor (TCR), CD3 is always selected as a cell surface target [13]. Thus, numerous bsAbs targeting CD3 are developed in a variety of different constructs, they can be broadly classified into two categories: bsAbs with Fc domains and bsAbs without Fc domains. Fc domains contribute to the maintenance of stability, simplification of the purification process, and extending of half-life for bsAbs [14]. However, the interaction between Fc domains and their receptors on various types of immune effector cells such as natural killer (NK) cells, monocytes, and macrophages, is capable of inducing antibody-dependent cell-mediated cytotoxicity (ADCC), while Fc domains can also bind complement to elicit complement-dependent cytotoxicity (CDC), leading to the unnecessary non-specific immune response during bsAbs treatment [15]. BiTE falls into the latter category with a small molecular size. Two single-chain variable fragments (scFvs) from an anti-TAA and an anti-CD3 monoclonal antibody, respectively, form the canonical BiTE molecule with a short linker connecting them in tandem [16]. scFvs consisting of heavy chain variable regions (VH) and light chain variable regions (VL) of immunoglobulin (Ig) are commonly used as antigen-binding domains (Fig. 1). Therefore, BiTE is a small and flexible molecule, which is easily diffusible and can quickly move from the site of administration to the site of lesions, redirecting cytotoxic T cells to cancer cells with high affinity.

Generally, TAAs are presented by major histocompatibility complex (MHC) molecules expressed on tumor cells, interacting with TCR on T cells and inducing T-cell activation to eliminate malignant cells, which are known as MHC restriction. However, the impairment or loss of the ability to present antigens for MHC molecules is one of the reasons for the intrinsic resistance to immunotherapies such as ICI therapy [17–19]. Moreover, effective T-cell activation and immune response also require costimulatory signals, primarily CD28 signals [20]. Nevertheless, BiTE can crosslink cytotoxic T cells and cancer cells independently from MHC restriction as well as costimulatory signals like a bridge, and then these T cells are activated and start to proliferate, inducing the formation of the immunologic synapse [21–23]. Perforins and granzymes are secreted by these activated T cells at the site of the immunologic synapse, which results in the cytotoxic lysis of target cells [24]. Compared with other bsAbs and monoclonal IgG antibodies, in cellular models, BiTE has a 100 ~ 10,000 fold higher efficacy in tumor cell lysis with a low ratio of T cells to target tumor cells [25]. In addition, BiTE can be produced by mammalian cell lines in large quantities, resulting in a relatively simple and fast production process compared with the lengthy and complicated culture of CAR T cells [16]. In the next section, we will present the latest clinical advances of BiTE therapy with published results in both hematologic neoplasms and solid tumors.

## Clinical advances of BiTE in hematological malignancies

A primary requirement for successful BiTE therapy is the identification of appropriate TAAs expressed on target cells other than normal cells to avoid on-target/off-tumor toxicity. Abnormal expression of CD19 can be observed in many hematologic malignancies associated with B cells, contributing to tumorigenesis, besides, expression of CD19 is restricted to malignant and normal B-lineage cells [26–28]. Thus, CD19 is an attractive target for immunotherapies against B-cell malignancies. Blinatumomab is the first-in-class CD19 × CD3 canonical BiTE construct with a low molecular weight of 55 kDa. While adults with newly diagnosed B-cell precursor acute lymphoblastic leukemia (B-ALL) have achieved a complete remission (CR) rate of 80–90% with chemotherapy [29], patients with relapsed or refractory (R/R) disease, the cytogenetic abnormality with Philadelphia (Ph) chromosome and the minimal residual disease (MRD) after initial therapy remain major challenges and are associated with poor prognosis. Moreover, R/R non-Hodgkin lymphoma (NHL) that most frequently originate from B cells is another challenge in treating patients with B-cell malignancies. Nevertheless, blinatumomab has demonstrated remarkable clinical efficacy in addressing these thorny issues, which is summarized by other recent reviews [3, 30, 31] (Table 2). Blinatumomab has been approved by FDA for the treatment of both children and adults with R/R B-ALL and gained accelerated approval to treat patients with MRD-positive B-ALL, which is the first and only BiTE antibody reaching the market. A phase 1 clinical trial evaluating next-generation BiTE with extended half-life (HLE) AMG 562 is ongoing in patients with R/R NHL (NCT03571828). Other main T-cell engaging bsAbs targeting CD19 and CD3 include TNB-486 [48], dual affinity retargeting antibody (DART) duvortuxizumab [49], and tandem diabody (TandAb) AFM11 [50] developed for B-cell malignancies. However, clinical trials for DART duvortuxizumab and TandAb AFM11 were discontinued due to severe toxicity during treatment.

**Table 2:** Clinical advances of blinatumomab in the treatment of B-cell malignancies

Study/NCT identifier	Phase	Indications	Number of patients	Blinatumomab dosing	Efficacy	Toxicity	Reference
GMALL (original study NCT00198991, follow-up study NCT00198978)	2	Adults (≥18 years) with MRD-positive B- ALL	21 (20 evaluable for response)	Continuous IV infusion at a dose of 15 μg/m^2^ /d	MRD-negative response rate: 80% (16/20) 5-year RFS rate: 50% (10/20) Median RFS: 19.1 months in 16 MRD responders; 3.2 months in 4 nonresponders CR in HSCT patients: 5/9 CR in non-HSCT patients: 5/11	Grade ≥ 3 AEs: 81% Grade 3 CNS AEs: 19%	[32–34]
BLAST (NCT01207388).	2	Adults with MRD-positive B-ALL	116 (113 evaluable for response)	Continuous IV infusion at a dose of 15 μg/m^2^ /d	MRD-negative response rate: 78% (88/113) 18 months RFS rate: 54% Median RFS: 18.9 months Median OS: 36.5 months 25% patients without HSCT after blinatumomab remained in continuous CR 49% with HSCT remained in continuous CR	Grade ≥ 3 AEs: 60% Any grade CRS: 3% Grade ≥ 3 CRS: 2% Any grade CNS AEs: 53% Grade ≥ 3 CNS AEs: 13%	[35, 36]
NCT02412306	1/2	Japanese adults with R/R Ph-negative B-ALL	26	Continuous IV infusion at a dose of 9 μg/m^2^ /d for the first 7 days and 28 μg/m^2^ /d thereafter	Phase 1b: CR/CRh rate: 80% (4/5) MRD-negative response rate in responders: 100% (4/4) Phase 2: CR/CRh rate: 38% (8/21) MRD-negative response rate in responders: 38% (3/8) Median RFS: 5 months	Grade ≥ 3 AEs: 96% Any grade CRS: 46% Grade ≥ 3 CRS: 4% Any grade CNS AEs: 46% Grade ≥ 3 CNS AEs: 5%	[37]
NCT01209286	2	Adults with R/R Ph-negative B-ALL	36	Continuous IV infusion at a dose of 5–30 μg/m^2^ /d	CR/CRh rate: 69%(25/36) MRD-negative response rate in responders: 88% (22/25) Median OS: 9.8 months Median RFS: 7.6 months	CNS AEs requiring intervention: 17% Grade ≥ 3 CRS: 6%	[38]
NCT01466179	2	Adults (≥18 years) with R/R Ph-negative B-ALL	189	Continuous IV infusion from 9 to 28 μg/m^2^/d	CR/CRh rate: 43%(81/189) MRD-negative response rate in responders: 82% (60/73) Median OS: 6.1 months Median RFS: 5.9 months	Grade ≥ 3 AEs: 83% Grade ≥ 3 CRS: 2% Any grade CNS AEs: 52% Grade ≥ 3 CNS AEs: 13%	[39]
TOWER (NCT02013167)	3	Adults (≥18 years) with R/R Ph-negative B-ALL	405 (271 for blinatumomab, 134 for chemotherapy)	Continuous IV infusion from 9 to 28 μg/m^2^/d	Median OS: 7.7 vs. 4.0 months CR rate: 34% vs. 16%; CR/CRh/CRi rate: 44% vs. 25% 6-month EFS: 31% vs. 12% Median DOR: 7.3 vs. 4.6 months	Grade ≥ 3 AEs: 87% vs. 92% Grade ≥ 3 CNS AEs: 9.4% vs.8.3% Grade ≥ 3 CRS: 4.9% vs. 0% (blinatumomab vs. chemotherapy)	[40]
RIALTO (NCT02187354)	Expanded Access	Children (≤17 years) with R/R B-ALL	110 (98 evaluable for response)	≤ 25% blasts: 15 μg/m^2^/d > 25% blasts: 5 μg/m^2^/d for first 7 days, 15 μg/m^2^/d thereafter,	CR rate: 59% (58/98) MRD-negative response rate in responders: 79% (46/58) Median OS:13.1 months Median RFS: 8.5 months 62% (36/58) proceeded to HSCT after achieving CR	Grade ≥ 3 AEs: 65% Grade ≥ 3 CRS: 3% Any grade CNS AEs: 42% Grade ≥ 3 CNS AEs: 5.5%	[41]
MT103–205 (NCT01471782)	1/2	Children (≤17 years) with R/R B-ALL	94 (70 evaluable for response)	Continuous IV infusion from 5 to 15 μg/m^2^/d	CR rate: 39% (27/70) MRD-negative response rate in responders: 52% (14/27) 48% (13/27) proceeded to HSCT after achieving CR	Grade ≥ 3 AEs: 87% Any grade CRS: 11% Grade ≥ 3 CRS: 6% Any grade CNS AEs: 24% Grade ≥ 3 CNS AEs: 4%	[42]
NCT02000427	2	Adults (≥18 years) with R/R Ph-positive B-ALL	45	Continuous IV infusion from 9 to 28 μg/m^2^/d	CR/CRh rate: 36%(16/45) MRD-negative response rate in responders: 88% (14/16) Median OS: 7.1 months Median RFS: 6.7 months 44% (7/16) proceeded to HSCT after achieving CR	Grade ≥ 3 AEs: 82% Any grade CRS: 7% Grade ≥ 3 CNS AEs: 7%	[43]
MT103–104 (NCT00274742)	1	Adults (≥18 years) with R/R B-NHL	76	Dose-escalation: 0.5–90 μg/m^2^/d Dose-expansion: 60 μg/m^2^/d	Patients with dose of 60 μg/m2/d: ORR: 69% across NHL subtypes and 55% for DLBCL Median DOR: 404 days Median OS in overall population: 4.6 years Median OS in responders: 7.7 years Median OS in nonresponders: 1.1 years	Grade 3, 4, and 5 AEs: 90, 66, and 4%, respectively Any grade CNS AEs: 71% Grade 3 CNS AEs: 22%	[44, 45]
NCT02910063	2	Adults with R/R B-NHL	41	Continuous IV infusion from 9 to 28 to 112 μg/d	ORR: 37% (15/41) including CMR: 22% (9/41) and PMR: 15% (6/41) HSCT was performed in 53.3% (8/15) responders.	Grade ≥ 3 AEs: 71% Grade ≥ 3 CRS: 2% Any grade CNS AEs: 56% Grade ≥ 3 CNS AEs.: 24%	[46]
NCT01741792	2	Adults with R/R B-DLBCL	25 (21 evaluable for response)	Cohort I + III: Stepwise dose 9 to 28 to 112 μg/d Cohort II: Flat dose 112 μg/d	Patients with dose of 112 μg/d at least 1 week: ORR: 43% (9/21) CR rate: 19% (4/21)	Cohort I: *n* = 23 Grade ≥ 3 AEs: 95.7% Any grade CRS: 0% Grade ≥ 3 CNS AEs: 22% Cohort II: *n* = 2 Grade ≥ 3 CNS AEs: 100%	[47]

*MRD* Minimal residual disease, *B-ALL* B-precursor acute lymphoblastic leukemia, *IV* Intravenous, *HSCT* Allogeneic hematopoietic stem cell transportation, *AEs* Adverse events, *R/R* Refractory/relapsed, *CRS* Cytokine release syndrome, *OS* Overall survival, *RFS* Relapse-free survival, *CR* Complete remission, *CRh* Complete remission with partial hematologic recovery, *CRi* Complete remission with incomplete hematological recovery, *Ph* Philadelphia chromosome, *NHL* Non- Hodgkin lymphoma, *DLBCL* Diffuse large B cell lymphoma, *DLT* Dose-limiting toxicity, *DOR* Duration of remission, *EFS* Event-free survival, *ORR* Objective response rate, *CMR* Complete metabolic response

CD33 mediates the proliferation and differentiation of myeloid cells and it can be detected on the malignant myeloid blasts in the majority of patients with acute myeloid leukemia (AML), suggesting it is a potential target for cancer immunotherapy [51]. In 2020 ASCO, updated data from an ongoing phase 1 study (NCT02520427) conducted for AMG 330, a CD33 × CD3 canonical BiTE antibody, has been reported. AMG 330 was given at doses ranging from 0.5–720 μg/d in the manner of continuous IV infusion among 55 patients with R/R AML. The most common treatment-related AEs was cytokine release syndrome (CRS) with a grade ≥ 3 CRS rate of 13%. CRS was reversible and was associated with the dose level and leukemic burden at baseline. Among 42 patients evaluable for response, there were 3 CR and 4 complete remission with incomplete hematological recovery (CRi) observed at the dose of ≥120 μg/d [52]. In addition, preliminary results from an ongoing phase 1clincal trial (NCT03224819) have shown anti-leukemic activity of HLE-BiTE AMG 673 in the treatment of R/R AML [53]. Currently, other main anti-CD33 T-cell engaging bsAbs have entered clinical development and are in phase 1 clinical study in patients with R/R AML, including JNJ-67571244 [54], affinity-tailored adaptors for T-cells (ATAC) GEM333 [55], and TandAb AMV 564 [56].

B-cell maturation antigen (BCMA) essential for the long-term survival of plasma cells is highly expressed on the malignant plasma cells in multiple myeloma (MM) with almost no expression on normal cells, which can be the promising target for T-cell based immunotherapy [57]. A first-in-human clinical trial recently demonstrated favorable efficacy and low toxicity of AMG 420, an anti-BCMA canonical BiTE molecule, treating patients with R/R MM. Forty-two patients who had previously been treated with more than 2 lines of therapies were enrolled and received 6-week cycles of AMG 420 at the dose of 0.2–800 μg/d. Among 10 patients in the maximum tolerated dose (MTD) cohort at the dose of 400 μg/d, the overall response rate (ORR) was 70% with 5 MRD-negative CR, 1 very good partial response (VGPR), and 1 PR. The serious AEs rate was 48%, of these, infections were the most common with a rate of 33%. The incidence of grade ≥ 3 CRS was 2% with no grade ≥ 3 central nervous system (CNS) toxicity [58]. AMG 420 has already been fast-tracked for development by the FDA. In addition, the second-generation HLE-BiTE AMG 701 has achieved an 83% ORR in patients with R/R MM, the majority of responders experienced triple therapy failure [59]. Teclistamab [60], REGN5458 [61], CC-93269 [62] PF-06863135 [63], and TNB-383B [64] are other investigational T-cell engaging bsAbs designed to bind to BCMA with structural differences, all of them have entered the early clinical stage for treating patients with R/R MM with promising efficacy. The clinical advances of these newly developed BiTE antibodies and other T-cell engaging bsAbs are summarized in Table 3.

**Table 3:** Clinical advances with promising published results in other BiTE and T-cell engaging bsAbs

NCT identifier	Phase	Indications	Number of patients	Drug	Format	Target	Results	Reference
NCT03625037	1/2	R/R B-NHL	67	Epcoritamab	Fully human IgG1-based bsAb	CD20/CD3	In DLBCL ≥12 mg (*n* = 18), ORR: 66.7% (6CRs) In FL ≥ 0.76 mg (*n* = 8), ORR:100% (2CRs) In MCL (*n* = 4) ORR: 50% (1 CR, 1 PR)	[65]
NCT02290951	1	R/R B-NHL	127	Odronextamab	Fully human IgG4-based bsAb	CD20/CD3	In FL ≥ 5 mg (*n* = 28), ORR: 92.9% (CR rate: 75.0%) In DLBCL without prior CAR T therapy ≥80 mg (*n* = 10), ORR: 60% (CR rate: 60%) In DLBCL with prior CAR T therapy ≥80 mg (*n* = 21), ORR: 33.3% (CR rate: 23.8%)	[66]
NCT02500407	1	R/R FL	62	Mosunetuzumab	Fully humanized IgG1-based bsAb	CD20/CD3	ORR: 68% (CR rate: 50%, PR rate: 18%)	[67]
NCT02924402	1	R/R NHL and CLL	44	XmAb13676	Fully humanized bsAb	CD20/CD3	In NHL 80–125 μg/kg (*n* = 18), ORR: 33.3%. In CLL 20 μg/kg (*n* = 5): ORR: 25%	[68]
NCT03075696	1	R/R NHL	38	Glofitamab	Fully humanized IgG1-based bsAb with a 2:1 molecular format	CD20/CD3	ORR:62.5%, CMR rate: 40.6% In aNHL (*n* = 24), ORR: 50.0%, CMR rate: 29.2%. In iNHL (n = 8), ORR: 100.0%, CMR rate: 75.0%	[69]
NCT04082936	1	R/R NHL	14	IGM-2323	Fully humanized IgM-based bsAb	CD20/CD3	Tumor size reduction rate: 64% (2 PRs)	[70]
NCT02520427	1	R/R AML	55	AMG 330	Two tandem scFvs	CD33/CD3	ORR: 19% (CR rate: 7%, CRi rate: 10%)	[52]
NCT03224819	1	R/R AML	30	AMG 673	Two tandem scFvs with Fc region	CD33/CD3	Decrease in blasts in bome marrow rate: 44%	[53]
NCT02514239	1	R/R MM	42	AMG 420	Two tandem scFvs	BCMA/CD3	ORR: 31% At dose of 400 mg/d (n = 10), ORR: 70% (5 MRD-negative CRs, 1 PR, and 1 VGPR)	[58]
NCT03287908	1	R/R MM	75	AMG 701	Two tandem scFvs with Fc region	BCMA/CD3	At the dose of 3–12 mg (*n* = 45), ORR: 36%; At the dose of the 9 mg (*n* = 6), ORR: 83% (3PRs, 2VGPRs)	[59]
NCT03145181	1	R/R MM	128	Teclistamab	Fully humanized IgG1-based bsAb	BCMA/CD3	At the dose of 1500 μg/kg sc (*n* = 22), ORR: 73% (≥ CR rate: 23% and ≥ VGPR rate: 55%)	[71]
NCT03761108	1	R/R MM	49	REGN5458	Fully humanized IgG1-based bsAb	BCMA/CD3	At the dose of 96 mg ORR: 63% (≥ VGPR rate: 95%)	[61]
NCT03269136	1	R/R MM	30	PF-06863135	Fully humanized IgG1-based bsAb	BCMA/CD3	At the dose of 215 to 1000 μg/kg SC (n = 20), ORR: 80% (6CRs, 3 VGPRs,6 PRs)	[72]
NCT03933735	1	R/R MM	38	TNB-383B	Fully humanized IgG1-based bsAb	BCMA/CD3	At the dose of ≥40 mg, ORR: 80% (VGPR rate: 75%)	[64]
NCT03275103	1	R/R MM	51	Cevostamab	Fully humanized IgG1-based bsAb	FcRH5/CD3	At the dose of ≥3.6-20 mg (*n* = 29), ORR: 51.7% (6 CRs, 4 VGPRs, and 5 PRs)	[73]
NCT03399799	1	R/R MM	137	Talquetamab	Fully humanized IgG4-based bsAb	GPRC5D/CD3	At the dose of 20–180 μg/kg IV (n = 18), ORR: 78% (6/6 responded at the 60 μg/kg) At the dose of 135–405 μg/kg SC (*n* = 12) ORR: 67% (3/4 responded at the 405 μg/kg)	[74]
NCT02152956	1/2	PIF and ER AML	38	Flotetuzumab	Two inter-exchange of Fv domains	CD123/CD3	CRR: 42.1% (7 CR, 4 CRh, 4 CRi, and 1 MLFS) In PIF AML (n = 24), CRR: 45.8% (5 CR, 3 CRh, and 3 CRi) In ER AML (*n* = 14), CRR: 35.7% (2 CR, 1 CRh, 1CRi and 1 MLFS)	[75]

*R/R* Refractory/relapsed, *IG* Immunoglobin, *bsAb* Bispecific antibody, *NHL* Non- Hodgkin lymphoma, *DLBCL* Diffuse large B cell lymphoma, *FL* Follicular lymphoma, *MCL* Mantle cell lymphoma, *ORR* Objective response rate, *CR* Complete remission, *CRh* Complete remission with partial hematologic recovery, *CRi* Complete remission with incomplete hematological recovery, *PR* Partial response, *VGPR* Very good partial response, *CMR* Complete metabolic response, *CAR* Chimeric antigen receptor, *CLL* Chronic lymphocytic leukemia, *aNHL* aggressive NHL, *iNHL* indolent NHL, *scFv* Single-chain fragment variable, *MRD* Minimal residual disease, *AML* Acute Myeloid Leukemia, *MM* Multiple myeloma, *Fc* Fragment crystallizable, *IV* Intravenous, *SC* Subcutaneous, *Fv* Fragment variable, *PIF* Primary induction failure, *LR* Late relapse, *MLFS* Morphologic leukemia-free state

## Clinical advances of BiTE in solid tumors

Given the favorable results of BiTE therapy in hematological malignancies, numerous BiTE antibodies targeting TAAs expressed on solid tumors are currently in clinical development (Table 4). Prostate-specific membrane antigen (PSMA) belongs to the type II transmembrane protein family highly confined to the prostate tissues. It is overexpressed in most prostate cancer (PC) and can be used as an ideal tumor antigen target [76]. Pasotuxizumab (AMG 212) is a canonical BiTE antibody targeting PSMA [77]. The anti-tumor activity which was defined by PSA level decrease was dose-dependent. Two patients achieved long-term PSA responses with more than 1 year of treatment at doses of 40 μg/d and 80 μg/d, respectively. The grade 3 AEs occurred in 81% of patients and the most common of them were decreased lymphocytes (44%) and infections (44%). Treatment-related SAE was observed in only one patient (fatigue). This is the first clinical study supporting BiTE immunotherapy’s role as an effective intervention in the treatment of patients with solid tumors. Moreover, in 2020 ESMO, HLE-BiTE AMG 160 has demonstrated a safety profile and showed evidence of preliminary efficacy in patients with mCRPC [78]. Another PSMA-targeting T-cell engager HPN424 has also entered a phase 1 clinical trial with early signs of clinical activity in patients with mCRPC [79].

**Table 4:** Ongoing clinical trials of BiTE and other CD3-targeting T-cell engager bsAbs in solid tumors

NCT	Phase	Indications	Number of patients	Drug	Target	Results	Reference
NCT01723475	1	Metastatic castration-resistant prostate cancer	47	Pasotuxizumab	CD3/PSMA	In SC group (*n* = 31), > 50% PSA decline rate:21% In cIV group (*n* = 16), > 50% PSA decline rate:19%	[77]
NCT03792841	1	Metastatic castration-resistant prostate cancer	43	AMG 160	CD3/PSMA	Any PSA decline rate: 68.6% > 50% PSA decline rate:34.3% In patients with evaluable disease (*n* = 15), there are 3 PRs and 8 SD	[78]
NCT03296696	1	R/R Glioblastoma	15	AMG 596	CD3/EGFRvIII	In patients with sufficient follow-up (n = 8), there are 1 PR and 2 SD	[80]
NCT03319940	1	R/R Small-cell lung cancer	40	AMG 757	CD3/DLL3	In patients with evaluable disease (*n* = 38), there are 6 confirmed PRs, 11 SD, and 1 unconfirmed PR	[81]
NCT04117958	1	Gastric cancer	–	AMG 199	CD3/MUC 17	Not reported	[82]
NCT04260191	1`	Gastric cancer	–	AMG 910	CD3/CLDN18.2	Not reported	[83]
NCT04128423	1	R/R solid tumors	11	AMV564	CD3/CD33	In patients with evaluable disease (*n* = 9), there are 1 PR and 4 SD	[84]
NCT02324257	1	CEA+ advanced solid tumors	80	Cibisatamab	CD3/CEA	In patients with mCRC at doses ≥60 mg (*n* = 36) Metabolic PR rate: 28%	[85]
NCT04501770	1	HER2+ advanced solid tumors	–	M802	CD3/HER2	Not reported	[86]
NCT04501744	1	Malignant ascites	–	M701	CD3/EpCAM	Not reported	[87]
NCT02748837	1	Solid malignancies	–	ERY974	CD3/GPC3	Not reported	[88]
NCT02248805	1	R/R Metastatic colorectal carcinoma	95	MGD007	CD3/gpA33	Not reported	[89]
NCT02262910	1	Metastatic castration-resistant prostate cancer	35	ES414	CD3/PSMA	Not reported	[90]
NCT03860207	1	R/R Neuroblastoma, osteosarcoma, and other GD2+ solid tumors	–	Hu3F8-bsAb	CD3/GD2	Not reported	[91]
NCT03983395	1/2	HER2+ metastatic breast cancers	–	ISB 1302	CD3/HER2	Not reported	–
NCT03448042	1	Locally advanced or metastatic HER2+ cancers	–	BTRC4017A	CD3/HER2	Not reported	–
NCT04424641	–	Locally advanced or metastatic solid tumors	–	GEN1044	CD3/5 T4	Not reported	–

*R/R* Refractory/relapsed, *PSMA* Prostate-specific membrane antigen, *cIV* Continuous intravenous, *SC* Subcutaneous, *PSA* Prostate-specific antigen, *CR* Complete remission, *PR* Partial response, *SD* Stable disease, *EGFRvIII* The epidermal growth factor receptor variant III, *DLL3* Delta-like 3, *MUC 17* Mucin 17, *CLDN18.2* Claudin-18 splice variant 2, *CEA* Carcinoembryonic antigen, *Her2* Human epidermal growth factor receptor 2, *EpCAM* Epithelial cellular adhesion molecule, *GPC3* Glypican 3, *gpA33* Glycoprotein A33, *GD2* Disialoganglioside

AMG 596 is a canonical BiTE molecule targeting the class III variant of the epidermal growth factor receptor (EGFRvIII). EGFR is a transmembrane cell surface receptor frequently overexpressed in patients with glioblastoma (GBM) that contributes to the formation of the tumor, meanwhile, EGFRvIII is the most common EGFR mutation in EGFR-positive patients with GBM [92]. Interim analysis of a phase 1 study (NCT03296696) evaluating the safety and efficacy of AMG 596 against recurrent GBM has shown 1 PR (12.5%) and 2 SD (25%) in 8 patients with sufficient follow-up. Among 14 evaluable patients, serious AEs occurred in 50% of patients, the most common treatment-related grade ≥ 3 AEs were headache and depressed consciousness (both 14.3%) [80].

Delta-like ligand 3 (DLL3) is particularly upregulated in small cell lung cancer (SCLC) but with minimal normal tissue expression. In 2020 the society for immunotherapy of cancer (SITC), preliminary results from an ongoing phase 1 study (NCT03319940) has demonstrated anti-tumor activity of AMG 757, a DLL3-targeting HLE-BiTE, in patients with R/R SCLC with tolerable toxicity [81]. Among 38 evaluable patients, there were 6 PR, 11 SD, and 1 unconfirmed PR. With a median follow-up of 8.8 months, five of the six responses were still in duration. The most common treatment-related AE was CRS with a rate of 43% and all CRS events were grade 1 or 2.

Additionally, AMG 199 [82] targeting mucin-17 (MUC17) and AMG 910 [83] targeting claudin18.2 (CLDN18.2) in patients with gastric cancer are in phase 1 clinical trials of NCT04117958 and NCT04260191, respectively.

## Predictors of response to BiTE therapy

Blinatumomab represents BiTE therapy because it the only one approved for clinical use with the most outcome data. Some studies have reported that the high disease burden defined as ≥50% bone-marrow blasts was associated with the lower response rate of blinatumomab in the treatment of R/R B-ALL [39, 40, 42, 93]. For instance, in the phase 3 study, the remission rate including CR with full, partial, or incomplete hematologic recovery was 65.5% in R/R B-ALL patients with less than 50% of bone-marrow blasts, whereas it was only 34.4% for ≥50% [40]. A recent statistical study confirmed the negative correlation between disease burden and treatment response was statistically significant in R/R B-ALL patients treated with blinatumomab (*P* = 0.039) [94]. Moreover, the negative correlations between disease burden and response rate were also observed in CAR T cell therapy [95]. Therefore, before the initiation of T-cell engaging therapies such as blinatumomab treatment, administration of cytoreductive therapies such as dexamethasone alone and chemotherapy combined with dexamethasone can be a reasonable strategy for improving response rates for patients with high disease burden [96]. However, it is noted that no standard measurement has been established for disease burden.

Extramedullary disease (EMD) can be regarded as the surrogate for disease burden to an extent, which is one of the known manifestations of advanced hematological malignancies. A retrospective study suggested that a history of the EMD at baseline, as well as the occurrence of EMD during blinatumomab treatment, was associated with lower CR rates in patients with R/R B-ALL (*P* = 0.005 and *P* = 0.05, respectively) [93]. Bone marrow MRD constitutes another surrogate for disease burden. A post hoc analysis evaluated several early parameters for predicting response to blinatumomab in children with R/R B-ALL and identified that day 15 bone marrow MRD could predict complete MRD response to blinatumomab with high accuracy of up to 95% within the first two treatment cycles [97]. On day 15 of treatment, 59 patients were assessed for MRD, among 46 MRD positive patients, 44/46 patients had no complete MRD response with an accuracy of 96%, meanwhile, 12/13 patients achieved complete MRD response with an accuracy of 92% for 13 MRD negative patients [97].

Both BiTE therapy and CAR T cell therapy are dependent on targeting TAAs on the tumor cell surface, leading to T cell activation, proliferation, and cytokine production. Thus the density of TAAs should be one of the key factors for effective anti-tumor activity which is associated with treatment efficacy [98]. A previous study suggested that the CD20 density was no less than 200 molecules per target cell for inducing CAR T cell-mediated cytolysis while ∼10-fold higher antigen density was required for cytokine production for an anti-CD20 CAR T cell construct in vitro experiments [99]. A quantitative systems pharmacology (QSP) model was developed to assess some other factors affecting blinatumomab anti-tumor efficacy in NHL patients, besides tumor antigen density, factors significant for anti-tumor efficacy were blinatumomab density, rate of redirected T cell lysis, tumor cell growth rate, and E/T ratio [100, 101]. Potential biomarkers for predicting response included tumor-infiltrating lymphocytes (TILs), primarily T cells, and tumor growth rate [101]. However, it should be noted that the number of regulatory T cells (Tregs) was inversely correlated with the response rate to blinatumomab in patients with R/R B-ALL [102]. Blinatumomab responders (*n* = 22) had an average Tregs of 4.82% (CI: 1.79–8.34%) in the peripheral blood, whereas it was 10.25% (CI: 3.36–65.9%) for non-responders (*n* = 20). All of the blinatumomab responders were successfully identified by the Treg cutoff of 8.525%, while 70% of the non-responders were excluded by this threshold.

The analysis of the relationship between tumor genetic features and treatment response is a useful way to select potential biomarkers for predicting outcomes of patients and guiding treatment decisions in the clinic. A recent study characterized the genetic profile in 44 adults with R/R B-ALL treated with blinatumomab to find out potential biomarkers to blinatumomab therapy [94]. Sixteen Ph-like patients with CRLF2 rearrangement had a higher response rate of 75%, whereas the response rate was 55% in the overall cohort. More importantly, the partial deletion of exon 2 of CD19, CD19 ex2part, was identified as a potential biomarker that predicted response to blinatumomab. In contrast to the responders, CD19 ex2part levels were significantly higher in blinatumomab-unresponsive patients by analyzing samples from pre-treatment patients (*P* = 0.025). Additionally, samples from post-treatment patients with relapse presented higher CD19 ex2part levels compared with those from pre-treatment patients, which indicated that CD19 ex2part accumulated during treatment and likely led to treatment failure (*P* = 0.0002) [94]. The predictive role of CD19 ex2part as a biomarker should be confirmed by future research for CD19-targeted immunotherapies, as well as exploring other potential biomarkers relative to TAAs.

## Toxicity

The safety profile of BiTE immunotherapy is of considerable concern to physicians and patients. As the only marketed member of BiTE family, the safety profile of blinatumomab is fully elucidated. In the phase 3 TOWER study, grade ≥ 3 AEs were ordered according to the frequency of occurrences, the top five were neutropenia, infection, elevated liver enzyme, neurotoxicity, and CRS [40]. Among them, the most concerning AEs are CRS and neurotoxicity which have proven dose-limiting toxicities (DLTs) and can even lead to death [103, 104]. CRS is an uncontrolled systemic inflammatory response with elevated levels of pro-inflammatory cytokines, primarily IL-6, which is triggered by T cell activation in T cell-engaging immunotherapies such as BiTE therapy and CAR T cell therapy [105]. CRS-related symptoms can range from mild flu-like symptomatology to severe and fatal multi-organ failure [105]. CRS often occurs in the first several days after BiTE infusion and the risk of grade ≥ 3 CRS in pivotal studies ranges from 0 to 6% for B-cell malignancies, in addition, a higher incidence of CRS is associated with higher tumor burden and drug doses [35, 38–40, 43, 46, 47]. The strategy of prophylactic use of dexamethasone combined with stepwise administration of blinatumomab is useful to decrease the risk of CRS [38]. After initiation of blinatumomab, interleukin (IL)-6 receptor inhibitor tocilizumab and corticosteroid are effective to treat severe CRS, while mild CRS can be addressed by symptomatic treatment [105].

Neurotoxicity is another unique treatment-related AEs for patients treated with T-cell engaging therapies, which is also termed as immune effector cell-associated neurotoxicity syndrome (ICANS). The incidence of grade ≥ 3 neurotoxicity ranges from 5.5 to 24% in these clinical studies related to blinatumomab [32, 35, 38–44, 47]. Neurotoxicity occurs mainly in treatment cycle 1 and the risk of neurotoxicity is increased when given higher doses of blinatumomab with the most common symptoms of dizziness, tremor, confusional state, and encephalopathy [104]. However, pathogenic mechanisms responsible for neurotoxicity induced by T cell-based anti-cancer therapies are complex and incompletely understood [106]. There was preclinical and clinical evidence that the adhesion of T cells to endothelial cells contributed to neurotoxicity induced by blinatumomab based on analyses of selected patients from 5 clinical trials and data from in vitro experiments [107]. Blinatumomab induced peripheral T-cell recruitment to the brain involving the following process: T cells adhered to cerebral microvascular endothelium, endothelial cells were activated with an increased level of Ang-2 (a marker of endothelial cell activation), T cells transmigrated across the blood-brain barrier into the brain. And then these T cells eliminated resident B cells with the release of cytokines, leading to an excessive immune response and ultimately resulting in neurotoxicity. The study suggested that agents inhibiting the adhesion between T cells and blood vessel endothelium could be an effective modality to reduce the incidence of neurotoxicity [107]. Interventions used routinely to treat neurotoxicity are interrupting treatment and dexamethasone [104].

To reduce minimize the risk of key systemic toxicity such as CRS, the safety, PK profile, and efficacy with subcutaneous (SC) administration of blinatumomab, a novel route of administration, is being evaluated in R/R B-ALL (NCT04521231) and R/R NHL (NCT02961881). In comparison to IV infusion, other potential benefits of SC injection include improved convenience and compliance and a reduction of cost for patients receiving treatment. Preliminary results regarding other bAbs that redirect T cells have been reported, suggesting SC injection did reduce the maximum concentration associated with CRS with high bioavailability, SC dosing is therefore a preferable route of administration for mitigating CRS and increasing the dose intensity [108, 109].

## Resistance to BiTE therapy

**Fig. 2:**
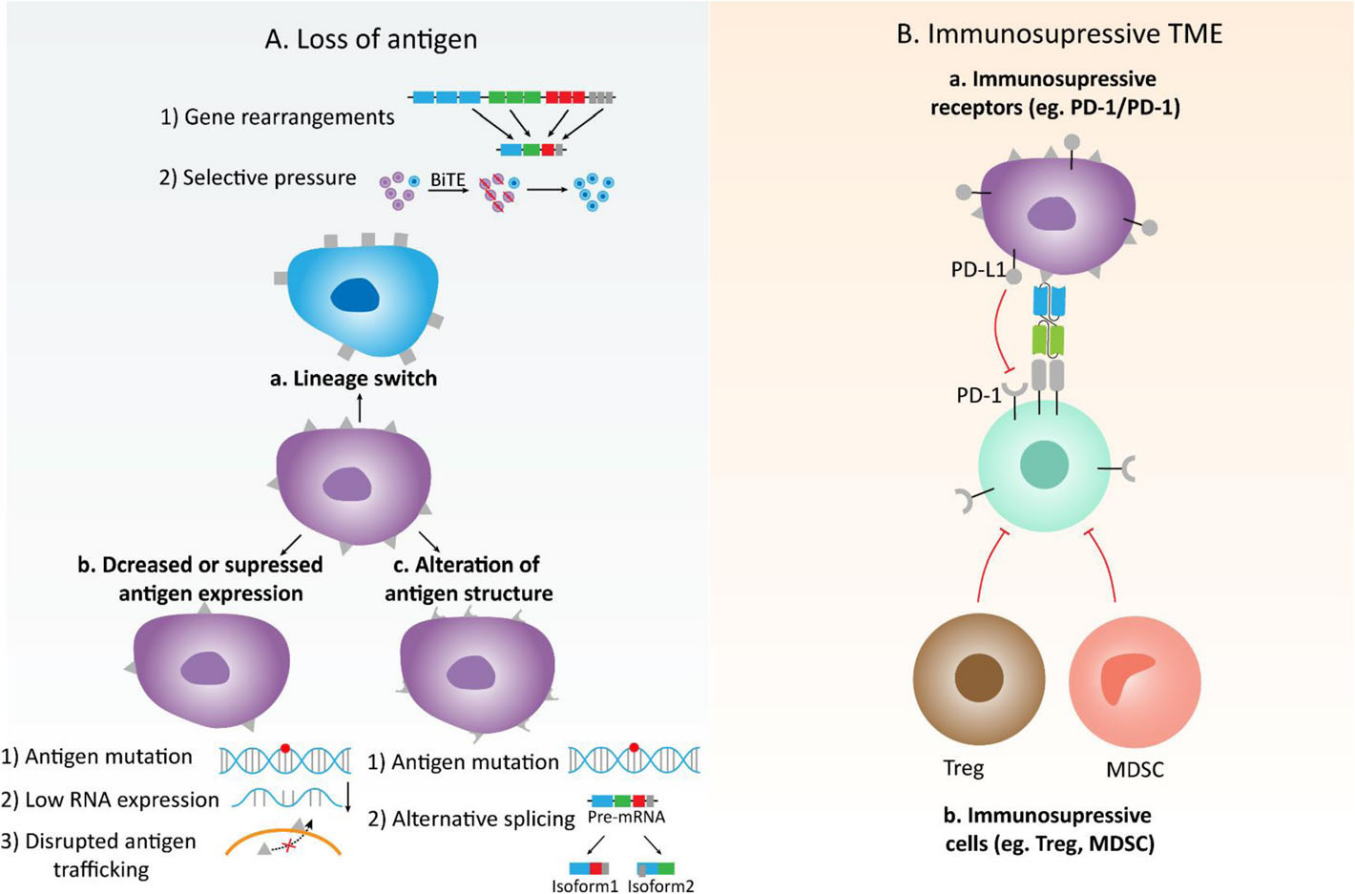
Summary of currently identified mechanisms of tumor evasion to BiTE therapy. **A.** Loss of antigen---possible mechanisms: a. Lineage switch associated with 1) Gene rearrangement 2) Selective pressure due to bispecific T cell engager (BiTE) therapy b. Decreased or suppressed antigen expression associated with 1) Antigen mutation 2) Low RNA levels 3) Disrupted antigen trafficking process c. Alteration of antigen structure associated with 1) Antigen mutation 2) Alternative spicing **B.** Immunosuppressive tumor microenvironment (TME)---possible mechanisms: a. Immunosuppressive receptors such as PD-1/PD-L1 axis b. Immunosuppressive immune cells such as T regular (Treg) cells and MDSCs: Myeloid-derived suppressor cells; PD-1: Programmed cell death 1; PD- L1: Programmed cell death 1 ligand 1

Despite the success of BiTE therapy with blinatumomab against B-cell malignancies expressing CD19, a significant portion of patients does not respond to treatment or they eventually relapse even with initial responses. The resistance to blinatumomab appears to involve multiple mechanisms, at present, studies have mainly focused on two aspects including immunosuppressive factors and loss of CD19 antigen (Fig. 2). Numerous studies have elucidated the key role of immune checkpoints in the suppression of anti-tumor immune responses, antibodies blocking the PD-1/PD-L1 axis have led to remarkable clinical efficacy for various malignancies [110]. Five years ago a case report described a patient with R/R B-ALL who experienced an increase in PD-1 and PD-L1 positivity after treatment with blinatumomab, in vitro experiments showed the ability of T cells inducing tumor cell lysis was weakened with a lower level of interferon γ (IFN-γ) in this patient [111]. Subsequent studies confirmed the finding that the expression of inhibitory immune checkpoints, mainly PD-L1, was increased after BiTE treatment in ex vivo cytotoxicity experiments and patients with different hematologic neoplasms, suggesting the addition of checkpoint inhibitors to BiTE therapy was a feasible strategy to improve BiTE-mediated cytotoxicity [100, 112]. Various solid tumor cells express immune checkpoint proteins on their surface that bind to inhibitory receptors on T cells, dampening the T-cell immune response and compromising the efficacy of T cell-based immunotherapies such as CAR T cell therapy and BiTE therapy [113]. Thus with the inhibition of immune checkpoints, the indications of BiTE therapy as well as CAR T cell therapy will expand including for both hematologic malignancies and solid tumors. The immunosuppressive cells in tumor microenvironment (TME) also present critical obstacles for effective immunotherapies, the most prominent representative is glioblastoma which is known to contain a dominance of immunosuppressive myeloid cells [114]. Tumors utilize Treg to suppress the tumor-specific immune response and promote their development, inhibition of blinatumomab efficacy by increasing levels of Treg measured by CD4/CD25/FOXP3 expression has been identified during blinatumomab treatment in patients with R/R B-ALL [102, 115]. Myeloid-derived suppressor cells (MDSCs), as another important component of immunosuppressive cells in TME, have received increasing attention in recent years that contribute to tumor development growth and progression [116]. A recent study identified neutrophils with the expression of CD11b/CD13/CD16 belonged to granulocytic MDSCs (G-MDSCs) in patients with MM, moreover, depleting these immunosuppressive cells could improve the cytotoxic activity of BCMA/CD3 bsABs [117]. Collectively, with the deep understanding of immunosuppressive factors in TME further related targeted therapies will combat resistance to current T-cell based immunotherapies.

Although B-ALL patients with initial CD19 positivity achieved CR after blinatumomab treatment, a considerable proportion of patients experienced relapse and CD19-negative relapse contributed to 8–50% of overall relapse [32, 33, 38, 42, 93, 118]. The targeted antigen loss was also observed in patients who were refractory to anti-CD19 CAR T cell treatment, suggesting the critical role of antigen loss in resistance to T-cell based cancer immunotherapies [119, 120]. Antigen loss can be interpreted as the loss of antigen expression and the loss of antigen-binding to targeted antibodies or cells, the presence of either situation or both can lead to the CD19-negative relapse. A study analyzed data from four B-ALL patients who had been treated with blinatumomab and experienced CD19-negative relapse and found that CD19 trafficking from the intracellular space to the membrane of B cells was prevented with the lack of CD81 that provided docking sites for CD19 signal transduction, resulting in absent CD19 expression [121]. CD19 mutations including frame-shift insertion or deletions, in-frame deletion, non-sense mutations, and splice-site single nucleotide variants (SNVs) were observed in five of seven R/R B-ALL patients who had CD19-negative relapse after blinatumomab treatment, suggesting CD19 mutations were common. Moreover, mechanisms of CD19 mutant allele-specific expression, low CD19 RNA expression, and mutations in CD81 which were in line with the previous study were also identified as partial causes of loss of CD19 expression [94]. Besides decreased or suppressed CD19 expression, notably, it was observed that usage of CD19 RNA isoform ex2part was increasing due to alternative splicing in both CD19-positive and CD19-negative relapses, which caused antigen escape by changing CD19 epitopes and ultimately disrupting the binding of blinatumomab to CD19 molecule rather than reducing CD19 expression [94]. One patient with CD19-negative relapse presented both antigen mutations and increased alternatively spliced RNA isoforms, suggesting they are likely to occur in parallel and responsible for antigen loss [94]. Alternative splicing and CD19 mutations were also demonstrated in other CD19-targeted immunotherapies such as CAR T cell therapy [122, 123]. Interestingly, the lineage switch of B lymphocytes from the lymphoid lineage to the myeloid lineage can be seen in patients with B-ALL who experienced CD19-directed immunotherapy in which CD19 expression was lost while myeloid marker levels such as CD33 were upregulated [124, 125]. The reasons for lineage switch may be associated with a strong selective advantage for subclones who did not express CD19 and gene rearrangements such as KMT2A/AFF1 and ZNF384 [126–128]. Therefore, multitargeted strategies may be effective to overcome antigen loss including developing a single drug that can simultaneously target dual or multiple TAAs or combining various immunotherapies that each of these targets a different TAA [129, 130].

## Development of novel T-cell engager antibodies

**Fig. 3:**
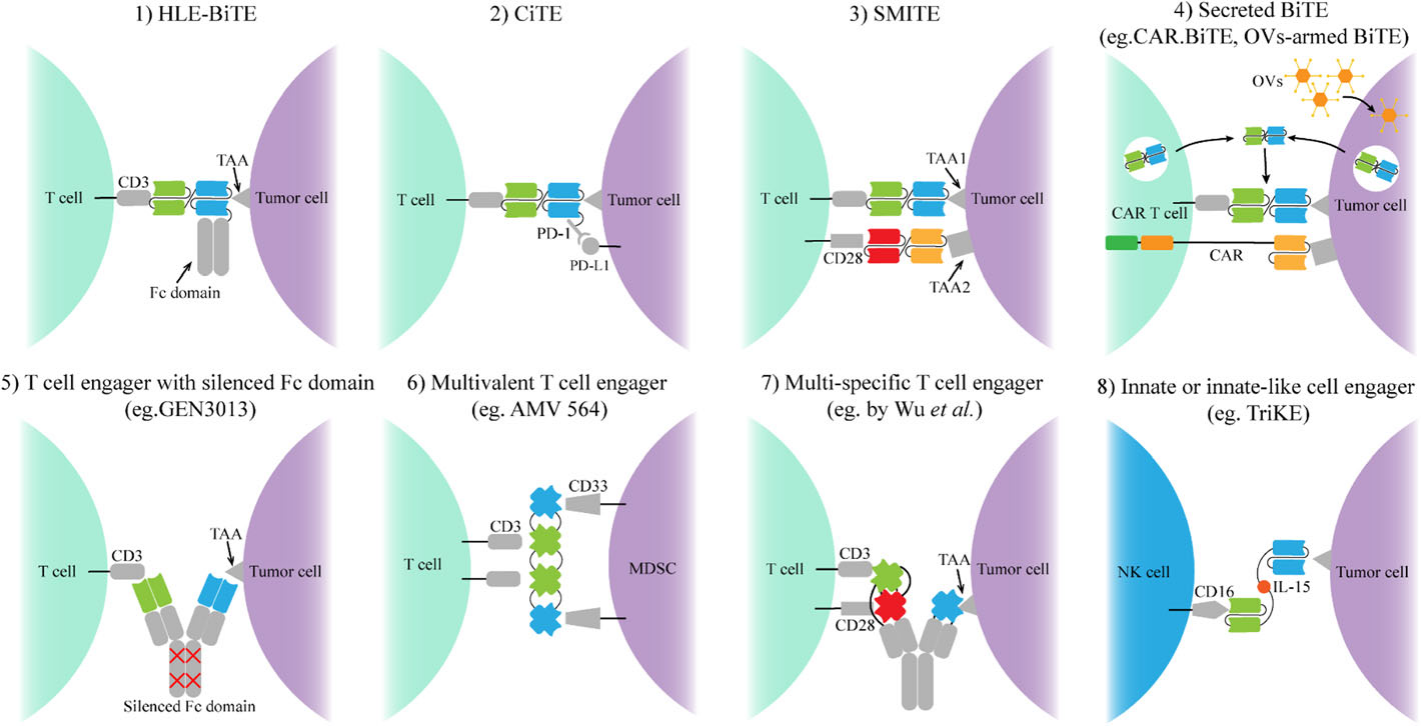
Schematic overview of the development of novel immune cell-based engager antibodies 1) Half-life extended-bispecific T cell engager (HLE-BiTE) 2) Checkpoint inhibitory T cell-engaging (CiTE) 3) Simultaneous multiple interaction T-cell engaging (SMiTE) 4) Secreted BiTE (eg. CAR.BiTE, OVs-armed BiTE) 5) T cell engager with silenced Fc domain 6) Multivalent T cell engager 7) Multi-specific T cell engager 8) Innate or innate-like cell engager CAR: chimeric antigen receptor OVs: Oncolytic viruses

Despite the clinical success of blinatumomab against B-cell malignancies, many obstacles remain such as inconvenient routes of administration, treatment resistance, and limited efficacy in solid tumors. To overcome these limitations, considerable efforts have been dedicated to structural modifying canonical BiTE construct and developing multifunctional T-cell engaging antibodies, while some of them have entered the clinical stage (Fig. 3).

### HLE-BiTE

Canonical BiTE antibody is composed of only two scFvs without the Fc domain that possesses the ability to extend circulation time, thus this small molecule can be quickly cleared through the kidney with a short half-life, which requires continuous intravenous infusion to maintain therapeutic serum concentrations [131]. To overcome this limitation and also to further reduce treatment cost, HLE-BiTE has been designed by adding an Fc domain to a BiTE molecule and patients require only a single administration per week [132]. However, there is concern that prolonged serum drug concentration may be associated with increased toxicity compared with canonical BiTE that quickly gets cleared by stopping the infusion. Clinical trials for a variety of HLE-BiTE antibodies are currently underway in both hematological malignancies and solid tumors, they have been summarized by the previous review [30]. For example, there are published preliminary data from a phase 1 first-in-human study for HLE-BiTE AMG 673, which targets CD33 in patients with R/R AML (NCT03224819) [53]. Thirty patients were enrolled and 67% of them had been treated with more than 4 anti-AML treatments. The mode of administration was a short IV infusion once a week in a two-week cycle, and patients were equally distributed in 10 cohorts with doses ranging from 0.05–72 μg. The median treatment cycle was 1.5. The incidence of treatment-related grade ≥ 3 AEs was 50% (15/30) including 5 abnormal hepatic enzyme, 4 CRS, 4 leukopenia, 2 thrombocytopenia, and 2 febrile neutropenia. The incidence of grade 3 CRS was 13.3%. Of the 27 evaluable patients, 11 (41%) presented a decrease in bone marrow blasts with one patient achieving a CRi at the dose of 36 μg [53]. Updated results showed 2 DLTs were observed in the additional dose cohort (110 μg), thus 110 μg was identified as MTD for further dose escalation [133]. Subsequent results from ongoing clinical trials for AMG 673 as well as other HLE-BiTE antibodies are expected.

### CiTE and SMiTE

It is established that the upregulation of immune checkpoints, such as PD-1/PD-L1, is one of the acquired resistance mechanisms to BiTE therapy [111]. The previous study found a significantly increased expression of PD-L1 when AMG 330 was added to AML cells in vitro, whereas immune blockade of the PD-1/PD-L1 interaction can greatly enhance the AMG 330-mediated cytotoxicity [100]. Thus, a novel bifunctional checkpoint inhibitory T cell-engaging (CiTE) antibody has been developed by fusing the PD-1 extracellular domain with low affinity to the canonical anti-CD33 BiTE construct [134]. Decreasing the affinity of the PD-1 extracellular domain to PD-L1 aims to restrict the development of immune-related AEs due to on-target off-leukemia toxicity, meanwhile, the affinity to CD33 tumor antigen is still high, which aims to maintain high specificity for CD33 + PD-L1+ double-positive cells other than all PD-L1+ cells. In vitro experiments, the CiTE antibody indeed showed a potent ability to activate T cells with an increased level of IFN -γ and cause cytotoxic lysis with high specificity for both CD33 + PD-L1 + cells and patient-derived AML cells [134]. CiTE can be a promising modified BiTE construct and is needed to be evaluated in more humanized models.

In general, activation of naive T cells requires at least two signals including TCR signal and costimulatory signal [135]. BiTE can generate TCR signal by binding to both CD3 and TAAs and form a powerful immunologic synapse between cytotoxic T cells and tumor cells, independent of the costimulatory signal [23]. However, a locally improved cytotoxic effect was observed in vitro and vivo studies when adding CD137, a potent costimulatory immunoreceptor, to BsAbs [136, 137]. Moreover, studies reported that activation of another costimulatory molecule CD28 enhanced the cytotoxicity of the BiTE antibody AMG 330 in primary human AML cells [138]. Thus, simultaneous multiple interaction T-cell engaging (SMITE) consisting of two separated canonical BiTE antibodies has been developed, both of the antibodies target TAAs on the one side while binding to either CD3 or CD28 on the other, providing additional costimulatory activation to enhance anti-tumor activity than either BiTE antibody alone [139]. In addition to targeting two different TAAs, one of BiTE antibodies can also target inhibitory immune checkpoints to overcome adaptive immune escape of tumor cells, mainly PD-L1, thus the SMITE pair was composed of CD3/TAA BiTE and CD28/PD-L1 BiTE [139].

### Secreted BiTE

The current delivery route for BiTE and other T cell engaging antibodies is through intravenous administration passively, while standard human antibodies are actively secreted by plasma cells under normal physiological conditions. If these therapeutic antibodies with a potent anti-tumor function are also secreted by living cells in the human body, it may maintain a stable therapeutic concentration and exert durable anti-tumor effects without continuous infusion, furtherly, BiTE-secreting cells should specifically target the tumor regions or these secreted cells themselves are even tumor cells to provide local production of BiTE antibody without increased systemic toxicity. Currently, CAR T cells and oncolytic viruses (OVs) are selected to express BiTE antibody by gene modification, which integrates two complimentary cancer immunotherapies into one vehicle that is expected to synergistically improve anti-tumor activity, especially in solid tumors for which the efficacy of either therapy alone is insufficient [140, 141]. In patients with GBM, although CAR-T cells targeting EGFRvIII decreased the number of EGFRvIII-expressing tumor cells, subsequent analysis of surgical samples showed a high level of wild-type EGFR in the tumor site. Therefore, Choi et al. proposed the concept of CART.BiTE and developed a kind of CART.BiTE product that was the special CAR-T cells targeting EGFRvIII that secreted EGFR-targeted BiTE antibodies. These CART.BiTE cells had effective anti-tumor activity against GBM with heterogeneous and even negative EGFRvIII expression in mouse models, precluding immune escape due to antigen loss with no toxicity observed in mice transplanted with human skin [140].

In recent years, BiTE and OVs have been merged into a single platform by arming OVs with a therapeutic transgene encoding BiTE, which is termed as BiTE-armed OVs. These modified viruses preferentially infect and replicate in tumor cells, ultimately inducing oncolysis, meanwhile, tumor cells infected with BiTE-armed OVs can trigger T-cell activation and lead to bystander killing of non-infected tumor cells, which greatly enhances anti-tumor activity than unmodified OVs [141]. By the use of OVs as BiTE gene delivery vehicles, BiTE can be secreted locally at the tumor site due to the tumor specificity of OVs, which minimizes BiTE-mediated systemic toxicity. In addition to targeting tumor cells, BiTE-armed OVs can be also used to target cells that promote tumor progression in TME such as immunosuppressive stromal cells and M2-like tumor-associated macrophages (TAMs) [142, 143]. Moreover, in addition to a gene encoding BiTE antibodies, OVs can be also equipped with expression cassettes encoding cytokine, checkpoint inhibitors, and other molecules that have effective anti-tumor activity. Recently, OVs that were capable of producing BiTE antibodies targeting TAA of CD44 variant 6 (CD44v6), interleukin-12 (IL-12, an immunostimulatory cytokine), and anti-PD-L1 antibodies were developed, while these multifunctional OVs in combination with CAR T cells targeting TAA of HER2 enabled a substantial improvement of disease control in CD44v6 positive tumors with heterogeneity HER2 expression, overcoming multiple resistance mechanisms of tumor antigen loss and inhibitory effect of immune checkpoints which are major obstacles for single immunotherapy in the treatment of solid tumors [144]. Consequently, integrating BiTE therapy with other bioactive cancer immunotherapies possessing tumor-targeting ability including CAR T cell and OVs therapy into one platform that enables actively secret BiTE antibodies is a promising research direction, retaining their respective advantages and compensating for their limitations to maximize therapeutic benefits while reducing BiTE-induced systemic toxicity.

### T cell engager with silenced Fc domain

The interaction between the Fc domain and its receptor on multiple immune cells can induce nonspecific immune activation such as ADCC, which leads to unwanted toxicity, however, the existence of the Fc domain enables antibodies to prolong the half-time. Thus, many antibodies with silent Fc domains have been developed by introducing point mutations that abrogate the binding of Fc receptors to Fc domains to reduce or avoid unnecessary effector functions while retaining the ability to extend the half-time [88, 145–151]. For example, GEN3013, a full-length human IgG1 bsAb recognizing CD3 and CD20, was generated by controlled Fab-arm exchange. Its Fc domain was silenced by the introduction of mutations L234F L235E D265A [151]. A phase 1/2 clinical trial is ongoing to evaluate the safety and efficacy of GEN3013 in patients with R/R B-cell lymphoma (NCT03625037). Moreover, recent studies have suggested that bsAbs with silenced Fc domains were able to improve the infiltration of T cells into solid tumors and enhance anti-tumor effects in the tumor xenograft mice, whereas bsAbs with intact Fc domains failed to induce T-cell trafficking to the tumor site because Fc-mediated effector functions enabled T cells to be sequestered in the reticuloendothelial system or depleted in circulation [152].

### Multivalent and multi-specific T cell engager

BiTE is a bispecific and bivalent antibody, therefore, it should be possible to enhance the anti-tumor activity and mitigating antigen loss by using multivalent and/or multi-specific T cell engager antibodies that carry multiple antigen-binding sites to increase binding affinity and/or recognize more different TAAs and molecules involving T-cell activation. Numerous multivalent T cell engager molecules are under active development and some of them have yielded promising results in early clinical trials [56, 62]. For instance, AMV 564, a tetravalent bispecific molecule, has 2 binding sites for CD3 on T cells and 2 binding sites for CD33 on MDSCs with 2:2 antigen-binding valency. MDSCs play an important role in mediating immune suppression and promoting tumor growth, which can serve as a novel target for many malignant diseases, thus, AMV 564 can eliminate immunosuppressive MDSCs and drive T cell activation and proliferation to effectively induce anti-tumor immunity [153]. In phase 1 first-in-human trial (NCT03144245), AMV564 was well-tolerated and effective in patients with R/R AML [56]. Moreover, interim results of an ongoing phase 1 study (NCT04128423) have shown that AMV 564 had anti-tumor activity in multiple solid tumors with tolerable toxicity [84]. Besides increasing antibody valency, developing multi-specific T-cell engager antibodies that direct at multiple molecules associated with anti-tumor immunity is intended to provide greater efficacy and overcome treatment resistance with lower toxicity. A trispecific T-cell engager antibody has been developed that simultaneously binds to CD3, CD28, and CD38, both CD3 and CD28 engagement resulted in superior T cell activation, while the recognition of CD38 redirects T cells against MM and other hematologic malignancies with powerful killing efficiency [154]. Although clinical trials regarding this trispecific antibody have not been performed in humans, the integration of multiple specificities into one protein is a promising next-generation T cell engager immunotherapy and deserves further investigation.

## Combination strategies

### Chemotherapy

Hyperfractionated cyclophosphamide, vincristine, doxorubicin, and dexamethasone (Hyper-CVAD) is a commonly used chemotherapy regimen for patients with newly diagnosed B-ALL, which can be served as a backbone for the development of new regimens [155]. A phase 2 study is evaluating the regimen of Hyper-CVAD in sequential combination with blinatumomab in adults with newly diagnosed B-ALL [156]. The regimen included 4 cycles of Hyper-CVAD followed by 4 cycles of blinatumomab. Twenty-seven patients were included, the median Hyper-CVAD treatment cycles were 3 while the median blinatumomab treatment cycles were 2, the rate of patients who underwent HSCT for high-risk features was 30%. The overall CR rate was 100% with an MRD-negativity response rate of 96%. With a median follow-up of 17 months, the 12-month RFS and OS rates were 76 and 89%, respectively. The incidence of blinatumomab-related grade ≥ 3 neurotoxicity was 17% while it was 4% for grade ≥ 3 CRS [156]. Two additional studies have also demonstrated the role of blinatumomab in combination with chemotherapy as front-line therapy in adults with newly diagnosed Ph- B-ALL with tolerable toxicity [157, 158]. In addition, blinatumomab monotherapy after current standard-of-care chemotherapy: a regimen of rituximab combined with cyclophosphamide, doxorubicin, vincristine, and prednisolone (R-CHOP) have achieved an ORR of 89% in patients with newly diagnosed high-risk DLBCL [159].

To further improve treatment outcome in patients with R/R disease, triple therapy based on the sequential addition of blinatumomab to the combination of low-intensity chemotherapy (mini-hyper-CVD) and inotuzumab ozogamicin, an anti-CD22 antibody-drug conjugate (ADC), has presented superior clinical benefits as the first salvage therapy compared to conventional intensive chemotherapy and single-agent inotuzumab ozogamicin in patients with R/R ALL with reducing the dose of inotuzumab ozogamicin and minimizing the risk of liver veno-occlusive disease (VOD) [160]. With a median follow-up of 3 years, the ORR was 80% with an MRD response rate of 83%, three-year CR duration and OS rates were 32 and 33%, respectively [161]. Numerous clinical studies evaluating the safety and efficacy of blinatumomab in combination with chemotherapy in the treatment of patients with newly diagnosed or R/R B-ALL are ongoing (Table 5).

**Table 5:** Ongoing clinical trials of blinatumomab in combination with other therapeutic approaches

Clinical Trials.gov identifier	Phase	Patient/Disease	Combination regimen	Class of combination drugs	Estimated enrollment	Primary endpoint	Status
NCT04554485	2	Adultswith newly diagnosed Ph-negative CD19+ B-ALL	Single cycle of blinatumomab followed by high-dose chemotherapy in the induction phase	Chemotherapy	45	CMR	Recruiting
NCT03518112	2	Adults with R/R Ph-negative B-ALL	Concurrent blinatumomab and low-intensity chemotherapy	Chemotherapy	44	EFS	Recruiting
NCT03480438	2	Older adults with newly diagnosed CD19+ Ph-negative B-ALL	Sequential dose reduced chemotherapy and blinatumomab	Chemotherapy	50	CHR and CMR	Recruiting
NCT02877303	2	Children and adults with newly diagnosed B-ALL	Sequential chemotherapy and blinatumomab	Chemotherapy	60	RFS	Recruiting
NCT03523429	2	Adults with high-risk CD19 + Ph-negative B-ALL	Blinatumomab added to consolidation phase with chemotherapy	Chemotherapy	38	MRD negative response	Recruiting
NCT03541083	2	Adults with newly diagnosed CD19+ B-ALL	Blinatumomab added to prephase and consolidation phase with chemotherapy	Chemotherapy	80	MRD negative response	Recruiting
NCT03367299	2	Adults with newly diagnosed or R/R CD19 + Ph-negative B-ALL	Sequential chemotherapy and blinatumomab	Chemotherapy	149	MRD negative response	Recruiting
NCT03914625	3	Children and adultswith newly diagnosed B-ALL	Addition of 2 cycles of blinatumomab to standard chemotherapy	Chemotherapy	6720 a	DFS	Recruiting
NCT02003222	3	Adults with newly diagnosed Ph-negative B-ALL	Sequential chemotherapy and blinatumomab	Chemotherapy	488	OS	Active, not recruiting
NCT02744768	2	Adults with newly diagnosed Ph + B-ALL	Sequential dasatinib and blinatumomab	TKI	60	MRD negative response	Recruiting
NCT02997761	2	Adults with R/R Ph-negative B-ALL	Concurrent Ibrutinib and blinatumomab	TKI	20	CR	Recruiting
NCT04530565	3	Adults with newly diagnosed Ph + B-ALL	Concurrent TKI and blinatumomab	TKI	330	OS	Recruiting
NCT04524455	1	Adults with R/R B-ALL	Blinatumomab in combination with AMG 404 (anti-PD-1)	ICI	21	DLTs and toxicity	Recruiting
NCT03605589	1	Children and adults with R/R CD19+ Ph-negative B-ALL	Blinatumomab in combination with pembrolizumab (anti-PD-1)	ICI	24	DLTs and toxicity	Suspended
NCT02879695	1	Children and adults with poor-risk R/R CD19+ B-ALL	Blinatumomab in combination with nivolumab with or without ipilimumab (anti-CTLA-4)	ICI	36	Toxicity and MTD	Recruiting
NCT03340766	1	Adults R/R DLBCL	Blinatumomab in combination with pembrolizumab	ICI	70	DLTs	Recruiting
NCT03160079	1/2	Adultswith R/R CD19+ Ph-negative B-ALL with high marrow lymphoblasts (≥50%)	Blinatumomab in combination with pembrolizumab	ICI	24	ORR	Recruiting
NCT03512405	1/2	Adultswith R/R CD19+ B-ALL	Blinatumomab in combination with pembrolizumab	ICI	36	Toxicity and ORR	Recruiting
NCT04546399	2	Children and adults with first relapse CD19+ B-ALL	Blinatumomab in combination with nivolumab (anti-PD-1)	ICI	550	MRD negative second response and EFS	Recruiting
NCT02143414	2	Older adults with newly diagnosed Ph-negative B-ALL (Cohort 1) and Ph + B-ALL (Cohort 2)	Blinatumomab followed by chemotherapy (Cohort 1) or dasatinib followed by blinatumomab (Cohort 2)	Chemotherapy or TKI	58	DLTs and OS	Recruiting
NCT03263572	2	Adults with Ph + B-ALL	Concurrent blinatumomab, chemotherapy, and ponatinib	Chemotherapy and TKI	60	CMR, ORR, RFS, EFS, and OS	Recruiting
NCT03147612	2	Adults with Ph + B-ALL	Sequential low-Intensity chemotherapy and ponatinib followed by blinatumomab and ponatinib	Chemotherapy and TKI	60	CMR and ORR	Recruiting
NCT02568553	1	Adults with R/R CD19+ B-NHL	Concurrent lenalidomide and blinatumomab	Others	44	Toxicity	Recruiting
NCT03751709	1	Adults (≥18 years) with R/R CD19+ B-ALL	Blinatumomab in combination with HLA-Mismatched Cellular Therapy	Others	10	DLTs	Recruiting
NCT03739814	2	Adults with newly diagnosed or R/R CD22+ Ph-negative B-ALL	Inotuzumab ozogamicin followed by blinatumomab	Others	64	EFS	Recruiting
NCT03982992	2	Adults with treatment-resistant MC or MRD of CD19+ B-ALL after HSCT	Blinatumomab in combination with donor lymphocyte infusion	Others	12	Toxicity	Recruiting

*Ph* Philadelphia chromosome, *CR* Complete remission, *CHR* Complete hematologic remission, *MRD* Minimal residual disease, *DLTs* Dose-limiting toxicities, *ORR* Overall response rate, *EFS* Event-free survival, *MTD* Maximum tolerated dose, *RFS* Relapse-free survival, *OS* Overall survival, *DFS* Disease free survival, *MC* Mixed chimerism, *DLBCL* Diffuse large B-cell lymphoma, *CMR* Complete molecular response, *HSCT* Allogeneic hematopoietic stem cell transportation

### Targeted therapy

Tyrosine kinase inhibitors (TKIs) targeting BCR-ABL1 are effective in the treatment of patients with Ph + leukemia, however, acquired resistance to TKI therapy remains a clinical challenge with limited therapeutic options. The retrospective study reported the encouraging efficacy of the combination strategy of blinatumomab and TKIs in patients with R/R Ph + B-ALL [162]. Among 16 patients with R/R disease, the CR rate was 88% with an MRD-negativity response rate of 86%. With a median follow-up of 15 months, the 12-month survival rate was 80% and the median OS following blinatumomab therapy was 45 months. Several other retrospective studies have also shown that blinatumomab combined with TKIs was safe and effective in patients with R/R Ph + disease [163, 164]. However, these studies are limited by retrospective design and small sample sizes, a prospective trial is investigating the efficacy of dasatinib, a second-generation TKI, in combination with blinatumomab as a front-line treatment in adults with Ph + ALL [165]. The regimen mainly consists of induction-phrase with dasatinib for 85 days and consolidation-phrase with blinatumomab. Among 63 patients treated with this regimen, the CR rate was 98%, the molecular response rate was 29.3% after completion of dasatinib-induction treatment and increased as the number of blinatumomab cycles increased (56.3% after 2 cycles of blinatumomab treatment, 65.7% after three cycles and 80% after 4 cycles). With a median follow-up of 10 months, the 12-month OS and disease-free survival (DFS) were 94.2 and 87.8%, respectively. Moreover, a recent case report has shown the combination of venetoclax, a selective inhibitor of the B-cell lymphoma 2 (Bcl-2), and blinatumomab is a highly effective and safe treatment against MRD relapse in patients with B-ALL [166]. Two patients achieved MRD-negativity responses soon after the complete blinatumomab treatment followed by venetoclax and underwent HSCT with no AEs. Several prospective trials to provide reliable evidence for this combination are ongoing (NCT02744768, NCT02997761, NCT04530565).

### Immune checkpoint inhibitors

The upregulation of immune checkpoints is one of the major mechanisms involved in resistance to BiTE therapy. The combination therapy with ICIs can reactivate an exhausted immune response and improve anti-tumor activity, thus several related clinical studies have been conducted. A small phase 1 study is evaluating the safety and tolerability of blinatumomab combined with nivolumab targeting PD-1 and/or ipilimumab targeting cytotoxic T-lymphocyte antigen 4 (CTLA-4) in patients with R/R B-ALL [167]. Eight patients were enrolled with the median BM blast percentage of 73%. Preliminary results have shown that the CR rate was 80% while all of them were MRD-negativity among 5 patients treated with blinatumomab and nivolumab. DLT was associated with a grade 4 infusion-related reaction. Another ongoing phase 1/2 study has also shown blinatumomab in combination with PD-1 inhibitor pembrolizumab is effective and safe in patients with R/R B-ALL and high disease burden at baseline [168]. The CR rate was 50% for four evaluable patients, while this study continues to evaluate efficacy by enrolling patients in the dose-expansion phase. Despite the impressive efficacy of ICI therapy against various solid tumors, the lack of tumor-reactive immune infiltration into tumors is one of the critical factors for the limited response rates. BiTE therapy can redirect large numbers of cytotoxic T cells to the tumor site to overcome this limitation and exert antigen-specific anti-tumor immunity. Furtherly, the therapeutic efficacy will be amplified by the addition of ICIs that facilitate a favorable immune profile and immune function of T cells by blocking immune checkpoint pathways.

Clinical trials concerning blinatumomab in combination with other therapeutic approaches involving immunomodulatory drug (lenalidomide), ADC (inotuzumab ozogamicin), and adoptive cellular therapy (donor lymphocyte infusion) are also in progress, which is summarized in Table 5.

## Beyond CD3 T cells

According to the diversity of expressed TCR, T cells are mainly divided into αβ and γδ T cells. Current cancer immunotherapies such as ICI therapy focus mostly on the MHC-restricted αβ T cells, while γδ T cells also present potent anti-tumor activity independently from MHC restriction, and thus immunotherapy based on Vγ9Vδ2 T cells, the predominant subtypes of γδ T cells, enable favorable clinical outcomes in various cancers with low toxicity [169, 170]. Bispecific γδ T cell engager is being developed, the antibody targeting Vγ9Vδ2 on γδ T cells and EGFR on tumors cells was demonstrated to induce strong activation of Vγ9Vδ2 T cells and cytotoxic lysis of tumor cells in a mouse xenograft model [171]. Further studies are needed to evaluate whether the BiTE for the recruitment of γδ T cells is more effective than classical BiTE targeting CD3 that is expressed on both αβ and γδ T cells [172]. In addition to T cells, NK cells are also essential effector cells as a part of innate immunity that contribute to anti-tumor responses, therefore, considerable interest has recently focused on the potential of NK cells for cancer immunotherapies [173]. Bispecific killer cell engager (BiKE) that ligate CD16 on NK cells and a tumor antigen on tumor cells has been developed to activate NK cells and induce target cell lysis, to further improve activation, expansion, and survival of NK cells, a modified IL-15 crosslinker is infused into BiKE to create trispecific killer engager (TriKE) which has shown superior anti-tumor activity compared with BiKE [174, 175] (Fig. 3). A phase1/2 clinical trial of GTB-3550 (CD16/IL-15/CD33) TriKE for the treatment of CD33-expressing high-risk myelodysplastic syndromes, R/R ALL, or advanced systemic mastocytosis is ongoing (NCT03214666). A new generation of trifunctional NK-cell engager (NKCE) targeting two activating receptors NKp46 and CD16 on NK cells and a specific antigen on tumor cells has been reported to have more potent anti-tumor activity than clinical therapeutic antibodies in vitro and in vivo [176]. Moreover, natural killer T cells (NKT) cells, a kind of innate-like lymphocytes, are quickly gaining attention as carries for CAR therapy termed as CAR-NKT cells which are currently being investigated in phase 1 clinical trial for patients with R/R neuroblastoma, preliminary results have shown CAR-NKT cells were safe and effective and can be applied to treat more patients (NCT03294954) [177]. Macrophages, another key component of innate immunity, can also be utilized as targeted immune effector cells in innate cell-based immunotherapies, the CAR macrophages (CAR-Ms) construct has been developed and has displayed excellent efficacy, reducing tumor burden and extending survival time in two solid tumor xenograft mouse models, as only one injection is needed [178]. Consequently, cancer immunotherapies for reactivating innate immunity hold great promise through targeting innate cells or innate-like cells such as NK cells, macrophages, NKT cells, and γδ T cells, complement or even replace current therapies.

## Conclusions

As the landscape of T cells and their role in anti-cancer immunity evolve, the development of T-cell-based immunotherapies has made a huge breakthrough in the last decade. In particular, the BiTE antibodies redirecting T cells to kill tumor cells have exhibited favorable clinical outcomes in R/R hematopoietic malignancies, as well as in solid tumors with preliminary evidence of clinical benefits. However, there remains a considerable number of patients that are resistant to the BiTE therapy and can not achieve durable responses. Current research has demonstrated that antigen loss and immunosuppressive factors, especially upregulation of inhibitory immune checkpoint molecules, are the two main mechanisms responsible for treatment failure. Therefore, strategies regarding improving BiTE constructs and developing novel T cell-engager antibodies with higher antigen avidities and multiple targets are currently being investigated in a series of preclinical and clinical studies, as well as combination therapies with BiTE antibodies and other therapeutic approaches. In addition to T cells, innate cell or innate-like cell engagers focusing on innate immunity are gaining increasing attention and have shown potent anti-tumor activity in various cancers, which reflects an orientation toward the future.

## Data Availability

Not applicable.
